# Idiopathic Lateral Rectus Myositis Without Signs of Orbital Inflammation

**DOI:** 10.7759/cureus.4859

**Published:** 2019-06-07

**Authors:** Mohammed Wazir, Mohammed FaisalUddin, Daniel Tambunan, Akriti G Jain

**Affiliations:** 1 Internal Medicine, Florida Hospital, Orlando, USA; 2 Internal Medicine, Deccan College of Medical Sciences, Hyderabad, IND

**Keywords:** pseudotumor, inflammatory pseudotumor, orbital tumors, orbital inflammation, idiopathic orbital inflammation

## Abstract

Idiopathic orbital inflammation (IOI), also known as orbital pseudotumor, is a nonspecific term that represents inflammation of unknown etiology that could affect various orbital structures. We report a case of IOI with an atypical presentation mimicking other clinical conditions. Our patient did not show the typical signs of inflammation that are usually seen in patients with orbital pseudotumor and are paramount in its diagnosis. Hence the diagnosis of IOI should be considered in the differential diagnosis of periorbital pain even when clinical signs of orbital inflammation are absent.

## Introduction

Idiopathic orbital inflammation (IOI) is a nonspecific term that represents inflammation of unknown etiology that could affect various orbital structures. IOI is the most common cause of painful orbital mass and can be localized to orbital adnexa or can be diffuse affecting orbital fatty tissues extending even to the cranium [1]. IOI can be unilateral or bilateral. It is the third most common disease of the eye and associated structures after Grave’s disease and lymphoproliferative disorders [2]. It is responsible for around 8%-11% of all orbital tumors [2]. IOI, however, is a diagnosis of exclusion, based on clinical picture, radiologic findings, and response to treatment. Here, we report a case of IOI with atypical presentation mimicking other clinical conditions.

## Case presentation

**Figure 1:**
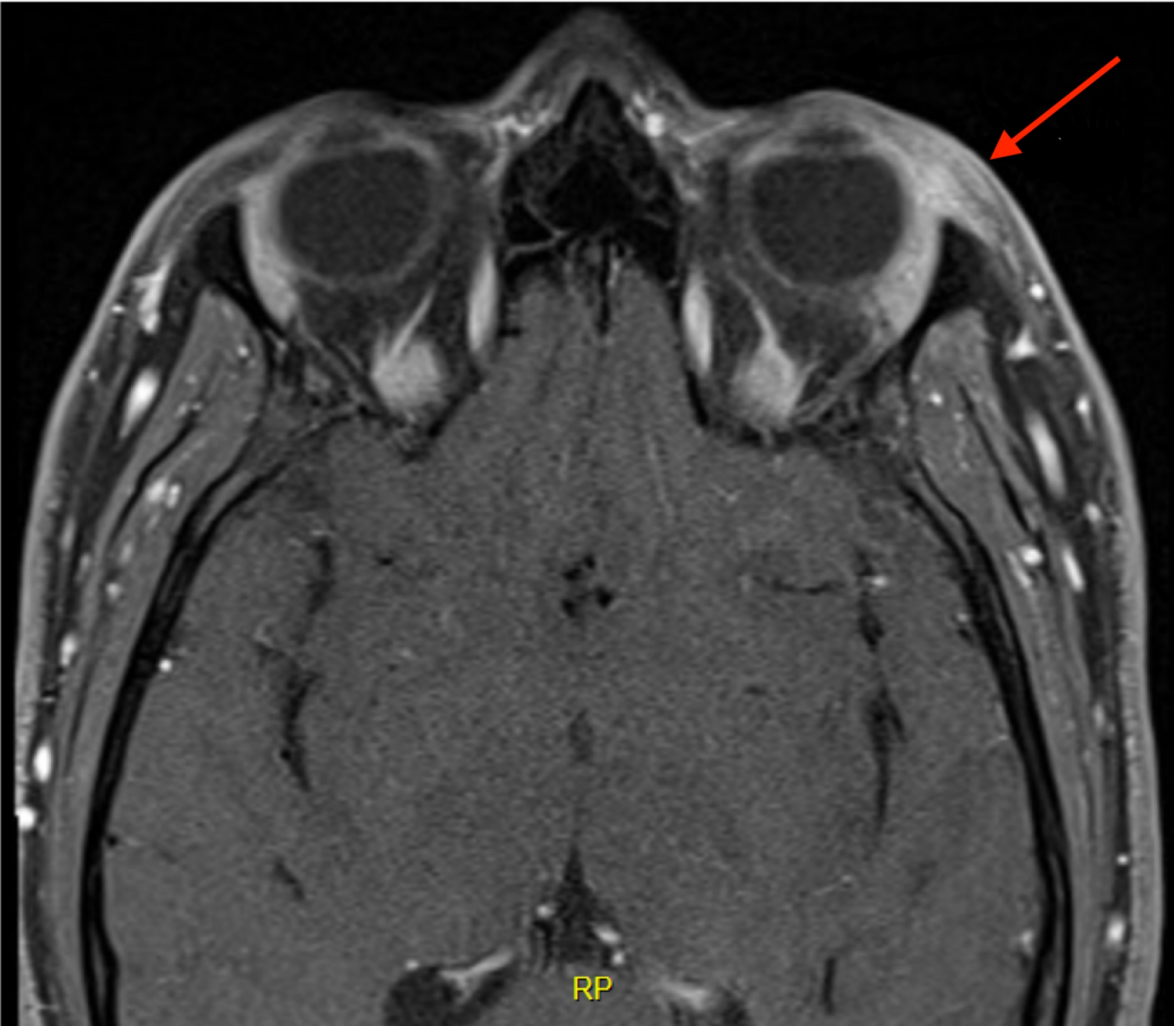
Orbital MRI showing increased enhancement of the left lateral rectus muscle (arrow).

An 18-year-old man presented to the ED complaining of pain in his left eye for four days. The pain was aggravated with horizontal eye movement, especially when looking to the left. There was no history of a blow or of other injuries to the orbit. The patient received treatment for sinusitis before presenting to the ED but his symptoms got worse. The patient was afebrile and vitally stable. On physical examination, abduction of the left eye was painful, but diplopia was not elicited in this direction of the gaze. There was also slight ptosis of the left eye at the time of initial presentation. Otherwise, there was no conjunctival injection, chemosis, proptosis, foreign body, or eye discharge. Visual acuity was 20/20 in both eyes. Visual fields, pupillary reactions, fundoscopic exam as well as the remainder of the neurologic and systemic examination were all normal. Results of laboratory investigations including complete blood count, basic metabolic panel, thyroid function tests, erythrocyte sedimentation rate, C-reactive protein, and anti-neutrophil cytoplasmic antibody were normal. Orbital and brain MRI showed enlargement with increased enhancement of the left lateral rectus muscle and the left lacrimal gland, with associated inflammatory changes in the adjacent left pre-septal soft tissues (Figure 1). There were also subtle inflammatory changes in the adjacent orbital fat. The cavernous sinuses were unremarkable bilaterally and no evidence of abnormality along the course of the cranial nerves. The patient was hospitalized and treated with high dose intravenous steroids. The response was rapid and dramatic with pain resolving completely within 24 hours from the onset of treatment. The patient was then treated as outpatient with oral prednisone slowly tapered over eight weeks with full remission and no complication or recurrence reported.

## Discussion

Idiopathic orbital inflammation, also known as orbital pseudotumor, is a benign nongranulomatous, noninfective, and nonneoplastic inflammatory process in the orbit in which a local or systemic cause cannot be established [3]. It was first characterised by Birch-Hirschfield in 1905 and was named idiopathic orbital inflammatory syndrome [4].

As IOI is a diagnosis of exclusion, exhaustive physical examination and clinical history is paramount with particular attention to rule out diseases associated with it including systemic immune-related diseases like orbital cellulitis, optic neuritis, thyroid ophthalmopathy, sarcoidosis, histiocytosis, Wegener’s granulomatosis, Tolosa Hunt syndrome, optic gliomas, lymphomas, and other neoplastic conditions [1]. Infection is a common cause of IOI which could be hiding in the structures around the orbit. Multiple orbital structures may be involved in this inflammatory process which includes the globe, the extraocular muscles, the lacrimal glands, the orbital fat sclera, the uvea, the superior orbital fissure, the cavernous sinus, and the optic nerve. Extra-orbital extension and bilateral eye involvement have also been reported [5].

Symptoms and presentation vary depending on the structure involved, degree of inflammation, fibrosis, entrapment, compression, and destruction of the adjacent structures [6]. Classical presentation usually is a clinical triad of periorbital pain especially with movement of the eye, ophthalmoparesis, and signs of inflammation such as redness, chemosis, proptosis, and periorbital edema. Other clinical features include diplopia, restricted motion, ptosis, and possible involvement of the optic nerve that can lead to blindness if left untreated.

Etiology of this disease is ambiguous which can be due to infectious process or immune mediated or both. A case of inflammation involving the orbital muscle has been reported right after a few weeks of confirmed streptococcal infection [7]. Immune-related etiology can be explained by its association with various immune disorders like systemic lupus erythematous, rheumatoid arthritis, ankylosing spondylitis, and Crohn’s disease [8-10]. Orbital contrast enhanced MRI is the single most important diagnostic test. Typical laboratory workup includes complete blood count, thyroid function test, anti-nuclear antibody (ANA) test, erythrocyte sedimentation rate, rheumatoid factor, and metabolic panel. Orbital imaging with CT scan or MRI may show signs such as uveoscleral thickening, optic nerve and extraocular muscle enlargement, contrast enhancement of the Tenon’s space with or without proptosis [2].

For mild disease observation is acceptable with or without nonsteroidal anti-inflammatory drugs (NSAIDS) like ibuprofen. For moderate to severe disease systemic steroids with slow tapering remains the mainstay of treatment [11]. Chemotherapy with methotrexate may be considered in some cases who are refractory or nontolerant to steroids [12-13]. Response to radiation therapy has also been documented [14]. A dramatic response to steroids is confirmatory and eliminates the need for invasive investigational procedures like orbital biopsy, as other conditions are not expected to exhibit such a dramatic and quick improvement. Our case was unique because the patient did not show characteristic signs of inflammation such as swelling and redness. Another important unique aspect in our case is the isolated involvement of the lateral rectus muscle. Orbital myositis is a subtype of IOI primarily affecting the extraocular muscles. The most frequently affected muscle is the inferior rectus. Involvement of lateral rectus muscle is considered rare. Periorbital pain with lack of such signs can mislead to a diagnosis of other conditions like abducens nerve palsy or paranasal sinusitis. 

## Conclusions

Idiopathic orbital inflammation should be considered in the differential diagnosis of peri-orbital pain even when other clinical signs of inflammation are missing. Following diagnosis, early initiation of systemic steroid therapy should ensue, which typically induces significant clinical improvement within few days of treatment.
